# Age- and Virus-Composition-Aware Interpretation of Respiratory Viral Co-Detection Using Same-Day C-Reactive Protein Profiles: A 2008–2024 Multiplex PCR Laboratory Cohort Study

**DOI:** 10.3390/microorganisms14071543

**Published:** 2026-07-15

**Authors:** Sung Hun Jang, Jeong Su Han, Bo Kyeung Jung, Ga-Yeon Kim, Jae Kyung Kim

**Affiliations:** 1 Department of Medical Laser, Graduate School of Medicine, Dankook University, Cheonan 31116, Republic of Korea; well8143@naver.com; 2 Department of Biomedical Laboratory Science, College of Health Sciences, Dankook University, Cheonan 31116, Republic of Korea; jshan1162@naver.com; 3 Department of Laboratory Medicine, College of Medicine, Dankook University, Cheonan 31116, Republic of Korea; lovegodmother@hanmail.net; 4 Department of Public Health, Dankook University Graduate School, Cheonan 31116, Chungnam, Republic of Korea; sysnhj77@gmail.com

**Keywords:** adenovirus, age group, C-reactive protein, diagnostic interpretation, host-response biomarker, multiplex PCR, respiratory viral co-detection, viral composition

## Abstract

**Objectives**: To determine whether patient age and viral composition provide more informative context than detected target number alone for interpreting same-day C-reactive protein (CRP) profiles when multiple respiratory viruses are detected. **Methods**: We retrospectively analyzed 19,002 same-day respiratory multiplex polymerase chain reaction (PCR)–serum CRP episodes collected at a Korean tertiary care hospital from October 2008 to December 2024. Episodes were classified as PCR-not-detected, single-virus detection, or viral co-detection. Among virus-positive episodes, associations with log-transformed CRP were evaluated using adjusted regression, leave-one-virus-out analyses, and a complete-panel mixed-effects model accounting for viral composition and repeated measurements. **Results**: Unadjusted CRP distributions were similar between single-virus detection and co-detection. After age- and sex-adjustment, co-detection was associated with 11.8% higher CRP. Excluding adenovirus-positive episodes attenuated the estimate to 2.7% (95% CI, −5.8 to 12.0; *p* = 0.541). In the complete-panel analysis of 1839 episodes from 1636 patients, co-detection was not independently associated with CRP, whereas adenovirus was associated with 75.1% higher CRP. Age group also showed strong graded associations with CRP levels overall. **Conclusions**: When multiple respiratory viruses are co-detected, same-day CRP profiles are better contextualized by patient age and the specific viral composition than by the number of detected targets alone.

## 1. Introduction

Multiplex polymerase chain reaction (PCR) has improved the detection of respiratory viral pathogens by enabling simultaneous identification of multiple viral targets in a single respiratory specimen. However, this increased diagnostic resolution has also made interpretation more complex, particularly when two or more respiratory viruses are detected in the same episode. Respiratory viral co-detection may reflect true concurrent infection, sequential infection with prolonged shedding, residual nucleic acid detection, or differences in viral replication dynamics. Accordingly, viral co-detection should not be interpreted as evidence of greater clinical severity or a proportionally increased systemic inflammatory response [1,2,3]. As the term “disease burden” is often used imprecisely, it is useful to distinguish between (i) clinical severity or symptom phenotype and (ii) inflammatory activity, which may be characterized using host-response biomarkers such as C-reactive protein (CRP) [4]. This distinction is clinically relevant because multiplex panels characterize pathogen detection, whereas CRP provides a readily available, nonspecific acute-phase signal reflecting one component of the systemic inflammatory response. CRP is widely used to support bacterial–viral discrimination as part of a broader clinical and laboratory assessment [5]. In this context, CRP serves as a complementary host-response readout alongside virological and clinical information [6]. This distinction is particularly important for multiplex PCR interpretation because concurrent CRP profiles may vary according to patient age, detected target number, and the specific viruses involved [7]. Respiratory viral co-detection represents a heterogeneous virological finding whose concurrent CRP profile may reflect both demographic context and viral composition. Our previous analysis of this laboratory cohort showed that age-related characteristics were more strongly associated with CRP variation than individual respiratory viral targets [8]. The present study addresses a distinct question: whether the number of simultaneously detected respiratory viral targets is associated with same-day CRP beyond patient age and the identities of the detected viruses. Patient age provides important interpretive context because both CRP profiles and respiratory virus distributions vary substantially across age groups. Viral composition provides an additional interpretive layer because individual respiratory viruses differ in their host-response associations. Adenovirus is particularly relevant in pediatric populations because adenoviral respiratory infection may be accompanied by relatively pronounced CRP elevation [9,10,11]. We therefore jointly evaluated the detected target number, age group, and virus-specific composition using leave-one-virus-out analyses and composition-adjusted repeated-measures models. This age- and virus-composition–aware approach was designed to determine whether target count alone adequately characterizes same-day CRP profiles in virus-positive multiplex PCR episodes.

## 2. Materials and Methods

### 2.1. Study Population and Data Source

This retrospective laboratory-based cohort study used respiratory multiplex PCR and serum CRP data obtained at Dankook University Hospital, a tertiary care hospital in the Republic of Korea. Eligible records were respiratory multiplex PCR tests performed from 2 October 2008 through 30 December 2024 that had a serum CRP result available on the same calendar day. The final analytical dataset comprised 19,002 PCR–CRP episodes. The dataset was extracted from the laboratory information system on 3 April 2026 and was fixed before statistical analysis.

### 2.2. Analytical Episode Definition

The analytical unit was a PCR–CRP episode, defined as one respiratory multiplex PCR result paired with the serum CRP value recorded in the same row of the laboratory dataset for the same calendar day. The dataset was constructed as a one-row-per-PCR–CRP-pair file before statistical analysis; therefore, the present analysis did not reselect CRP values according to within-day sampling time. When a patient underwent more than one eligible PCR test during the study period, each eligible PCR–CRP pair was retained as a separate episode to reflect the test-level structure of the laboratory dataset. To account for the correlation of repeated episodes within the same patient, a patient-level random intercept was included in the mixed-effects analysis. PCR results were categorized into three mutually exclusive detection groups: no respiratory virus detected (PCR-not-detected), single-virus detection, and viral co-detection. Single-virus detection was defined as the detection of exactly one respiratory viral target, whereas co-detection was defined as the detection of two or more respiratory viral targets in the same respiratory specimen. Virus-positive episodes consisted of both single-virus detection and co-detection episodes. PCR-not-detected episodes were used to characterize the overall tested population but were not included in the primary comparison between single-virus detection and co-detection.

For virus-specific descriptive analyses, episodes involving viral co-detection were included in the summary statistics for each detected viral target. Therefore, the resulting virus-specific descriptive summaries were not mutually exclusive. In contrast, regression analyses retained the episode-level structure of the data and incorporated co-detection status and virus-specific indicators as episode-level variables.

### 2.3. Respiratory Virus Testing and Target Availability

Nasopharyngeal swab specimens were used for respiratory virus testing. Specimens were processed according to the routine procedures of the clinical laboratory; when immediate processing was not possible, specimens were stored at 4 °C and analyzed within 24 h. Viral nucleic acids were extracted using the QIAamp Viral RNA Mini Kit (QIAGEN, Hilden, Germany) according to the manufacturer’s protocol.

Respiratory viral targets were detected using multiplex PCR platforms used in routine clinical practice during the study period. From 2008 to 2012, testing was performed using the Seeplex Respiratory Virus series (Seegene, Seoul, Republic of Korea), which was based on end-point PCR with gel electrophoresis. From 2013 onward, respiratory virus detection was performed using AdvanSure RV and RV-Plus real-time PCR/reverse-transcription PCR assays (LG Chem, Seoul, Republic of Korea) on the SLAN real-time PCR system (LG Life Sciences, Seoul, Republic of Korea).

As viral target availability changed during the study period, virus-specific results were interpreted according to the period in which each target was available in the analytical dataset. Human coronavirus NL63 and human bocavirus results were available from 12 January 2015 through 30 December 2024, whereas enterovirus results were available from 25 June 2018 through 30 December 2024. The remaining 12 respiratory viral targets were available throughout the analytical dataset. Accordingly, the complete-panel period, during which results for all 15 viral targets were available, was defined as 25 June 2018 through 30 December 2024. Target results that were unavailable before the introduction of a given assay were retained as missing and were not treated as negative detections. SARS-CoV-2 was not included among the respiratory viral targets evaluated in the present dataset. In the regression framework, virus-specific detection indicators were included to separate the association of co-detection status from the contributions of individual viral targets.

### 2.4. Serum CRP Measurement and Data Handling

Serum CRP was measured using an automated immunoturbidimetric assay on the Cobas 8000 modular analyzer with Tina-quant C-Reactive Protein Gen.3 reagents (Roche Diagnostics, Mannheim, Germany). Although the manufacturer’s analytical information is expressed in mg/L, CRP results in the institutional laboratory information system were recorded in mg/dL; therefore, mg/dL was used consistently in all analyses.

The reportable range in the laboratory dataset was 0.03–70 mg/dL. Results from 0.03 to 35 mg/dL were reported from direct measurement, whereas values > 35 mg/dL were obtained after automated 1:2 dilution and reported up to 70 mg/dL. The values recorded in the laboratory information system were analyzed without additional recalculation or substitution. Serum samples were processed according to routine laboratory procedures, including clotting, centrifugation, storage at 2–8 °C, and analysis within 24 h. Internal and external quality control procedures were maintained as part of routine laboratory practice.

### 2.5. Analytical Framework

The primary objective was to determine whether co-detection status retained an association with same-day CRP after patient age and virus-specific composition were accounted for. The analysis proceeded in three stages. First, CRP levels were compared between single-virus detection and co-detection among virus-positive episodes to characterize the count-based association. Second, age group and sex were incorporated to evaluate the demographic context of this association. Third, virus-specific CRP distributions, leave-one-virus-out analyses, and mixed-effects models were used to evaluate viral composition while accounting for repeated measurements within patients. Age-group and virus-specific estimates from the complete-panel mixed-effects model were interpreted together to characterize the contributions of demographic context and viral identity. CRP was treated as a nonspecific concurrent host-response measure used to contextualize, rather than determine, the significance of multiplex PCR detection patterns.

### 2.6. Statistical Analysis

Owing to the right-skewed distribution of CRP values, descriptive comparisons were based on nonparametric summaries. CRP concentrations are presented as medians and interquartile ranges. The Wilcoxon rank-sum test was used to compare CRP distributions between single-virus detection and co-detection groups.

As all CRP values were positive within the reportable analytical range, CRP values were natural-log transformed for regression analyses; in the analytical code, log(CRP + 0.01) was used to preserve consistency across all regression procedures. Linear regression models were fitted among virus-positive episodes to estimate the association between co-detection and CRP. Models were first fitted without covariate adjustment and then adjusted for age group and sex to quantify how demographic context shaped the estimated association between co-detection and CRP. Age was categorized as <1, 1–12, 13–18, 19–64, and ≥65 years and was interpreted as a clinically relevant contextual predictor of CRP. Ages recorded in months were converted to fractional years and classified in the <1-year age group.

Virus-specific descriptive comparisons were performed by comparing CRP levels between single-virus detection and co-detection for each respiratory viral target. *p*-values from these comparisons were adjusted using the Benjamini–Hochberg false discovery rate (FDR) procedure. Virus-specific CRP patterns were summarized using medians and interquartile ranges and visualized on a logarithmic scale.

Sensitivity analyses were conducted using leave-one-virus-out regression models. In each model, episodes positive for one viral target were excluded, and the association between co-detection and log-transformed CRP was re-estimated after adjustment for age group and sex. This approach was used to evaluate whether the overall co-detection association was disproportionately influenced by any specific virus.

To account for repeated testing and viral composition simultaneously, a linear mixed-effects model was fitted among virus-positive episodes collected from 25 June 2018 through 30 December 2024, corresponding to the period in which results for all 15 respiratory viral targets were available in the analytical dataset. Episodes collected before complete target availability were excluded from this analysis, and unavailable target results were not recoded as negative. The model simultaneously included age group, sex, co-detection status, and all 15 virus-specific detection indicators as fixed effects, with a patient-level random intercept. This complete-panel mixed-effects model was used to estimate whether co-detection was associated with CRP beyond the additive contributions of the individual detected viruses while accounting for repeated episodes within patients. Regression coefficients were back-transformed and reported as adjusted percentage changes in CRP with 95% confidence intervals. *p*-values were calculated using the Wald normal approximation. Benjamini–Hochberg FDR-adjusted *p*-values were additionally calculated across the 15 virus-specific coefficients. As an additional full-period clustering sensitivity analysis, generalized estimating equation models were fitted among virus-positive episodes using an exchangeable working correlation structure and patient identifier as the clustering variable. The analysis was restricted to virus-positive episodes defined using the 12 viral targets available throughout 2008–2024. In this sensitivity analysis, both virus-positive status and co-detection status were defined using only these 12 consistently available targets. Models included co-detection status, age group, sex, and virus-specific indicators. The analysis was repeated after excluding adenovirus-positive episodes. To evaluate the robustness of the findings to temporal changes in assay platform and the COVID-19 pandemic period, three additional sensitivity analyses were performed. First, the full-period GEE analysis restricted to the 12 viral targets available throughout 2008–2024 was additionally adjusted for assay platform and pandemic period. The assay platform was categorized as the Seeplex period (2008–2012) or the AdvanSure period (2013–2024), whereas the pandemic period was categorized as pre-pandemic (2008–2019), pandemic (2020–2022), or post-pandemic (2023–2024). Second, the analysis was restricted to the AdvanSure platform period and adjusted for the pandemic period. Third, the complete-panel mixed-effects model was additionally adjusted for the pandemic period. Regression coefficients were back-transformed and presented as adjusted percentage changes in CRP with 95% confidence intervals. All statistical tests were two-sided, and *p* < 0.05 was considered statistically significant. Analyses were performed using R software (version 4.3.3).

## 3. Results

### 3.1. Study Population and Respiratory Multiplex PCR Detection Categories

**Figure 1:**
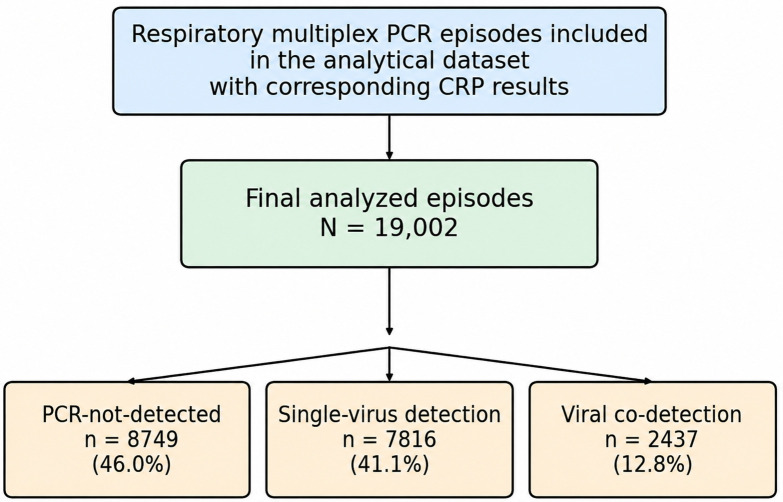
Classification of PCR–CRP episodes according to respiratory multiplex PCR detection status.

The classification of the study population is presented in Figure 1. A total of 19,002 episodes were included in the analysis. The median age was 3 years (IQR, 0–44 years), with an overall range of 0–98 years. The sex distribution was 11,338 males (59.7%) and 7664 females (40.3%). According to the detection category, 8749 episodes (46.0%) were PCR-not-detected, 7816 (41.1%) were single-virus detections, and 2437 (12.8%) were viral co-detection episodes; the total number of virus-positive episodes was 10,253. Age distributions differed markedly across detection categories. Children aged 1–12 years accounted for 46.6% of single-virus detection episodes and 64.1% of co-detection episodes, whereas adults aged ≥65 years accounted for 7.4% and 2.3%, respectively (Table S6).

Rhinovirus was the most commonly detected virus, accounting for 3559 episodes (18.7%), followed by adenovirus (1834 episodes; 9.7%), RSV A (1460 episodes; 7.7%), RSV B (1258 episodes; 6.6%), and influenza A (962 episodes; 5.1%). These were followed by parainfluenza virus type 3 (904 episodes; 4.8%), human metapneumovirus (802 episodes; 4.2%), OC43 (437 episodes; 2.3%), bocavirus (402 episodes; 4.0% of 10,140 specimens tested from 12 January 2015 through 30 December 2024), parainfluenza virus type 1 (383 episodes; 2.0%), influenza B (297 episodes; 1.6%), enterovirus (279 episodes; 5.0% of 5583 specimens tested from 25 June 2018 through 30 December 2024), coronavirus 229E (269 episodes; 1.4%), parainfluenza virus type 2 (130 episodes; 0.7%), and NL63 (126 episodes; 1.2% of 10,140 specimens tested from 12 January 2015 through 30 December 2024) (Table S1).

### 3.2. Age Distribution Shapes the Association Between Co-Detection and CRP

In the unadjusted comparison, CRP distributions did not differ statistically between the single-virus detection and co-detection groups (Table 1). Unadjusted regression also showed no statistical association between co-detection and CRP.

After adjustment for age group and sex among virus-positive episodes, co-detection was associated with an 11.8% higher CRP level than single-virus detection (95% CI, 4.3–19.9; *p* = 0.002), compared with an unadjusted estimate of +1.8%. Co-detection episodes were concentrated in younger age groups, in whom CRP values were generally lower than in older age groups. The change from the unadjusted to the age- and sex-adjusted estimate demonstrates that age distribution materially shaped the apparent count-based association. This age-adjusted estimate was subsequently evaluated together with virus-specific composition in the sensitivity and mixed-effects analyses.

### 3.3. Virus-Specific C-Reactive Protein Patterns Indicate Composition-Dependent Heterogeneity in Viral Co-Detection Episodes

Virus-specific comparisons showed marked heterogeneity in CRP patterns between single-virus detection and co-detection (Figure S1; Table S2). Adenovirus showed a lower median CRP in adenovirus-containing co-detection episodes than in adenovirus single-virus detection episodes (1.09 vs. 1.86 mg/dL; FDR-adjusted *p* < 0.001), whereas RSV B showed a higher median CRP in RSV B-containing co-detection episodes than in RSV B single-virus detection episodes (0.58 vs. 0.44 mg/dL; FDR-adjusted *p* = 0.008). In contrast, rhinovirus showed minimal difference between rhinovirus single-virus detection and rhinovirus-containing co-detection episodes (0.77 vs. 0.73 mg/dL; FDR-adjusted *p* = 0.385).

Among exact co-detection combinations, the most frequent combinations were rhinovirus + adenovirus, parainfluenza virus type 3 + rhinovirus, rhinovirus + enterovirus, RSV A + rhinovirus, and RSV B + rhinovirus, with median CRP values varying across combinations (Table S3). All 2437 co-detection episodes had at least two identifiable virus-specific positive indicators and were included in the exact combination-level summary.

### 3.4. Virus-Specific Heterogeneity of C-Reactive Protein Profiles According to Detection Status

**Figure 2:**
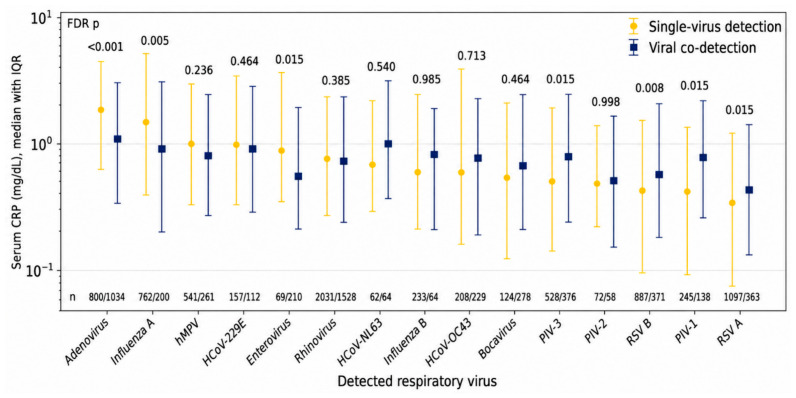
Virus-specific CRP profiles according to single-virus detection and co-detection status. Circles represent single-virus detection, and squares represent viral co-detection. Points indicate median CRP concentrations, and vertical bars indicate interquartile ranges. Values below the *x*-axis indicate the number of single-virus detection and co-detection episodes for each virus. Values above the plot indicate Benjamini–Hochberg false discovery rate (FDR)-adjusted *p*-values for comparisons between single-virus detection and co-detection. CRP concentrations are shown on a logarithmic scale. Virus-specific summaries are descriptive and non-mutually exclusive because co-detection episodes contributed to each detected viral target.

Figure 2 summarizes virus-specific CRP heterogeneity across detected respiratory viruses. Median CRP values and interquartile ranges differed by viral target and detection status, indicating that viral co-detection was not associated with a uniform shift in CRP across viruses. Adenovirus showed lower median CRP in co-detection than in single-virus detection, whereas RSV B showed higher median CRP in co-detection; rhinovirus showed minimal difference despite its large sample size (Table S2).

### 3.5. Joint Evaluation of Age and Viral Composition Refines the Interpretation of Co-Detection–Associated CRP Profiles

The descriptive and model-based analyses addressed different estimands: within-virus CRP distributions by detection status versus adjusted associations of co-detection and virus-specific indicators across virus-positive episodes. Adenovirus was markedly over-represented among co-detection episodes compared with single-virus detection episodes (1034 of 2437 (42.4%) vs. 800 of 7816 (10.2%)). Because adenovirus was independently associated with higher CRP in the composition-adjusted model, this differential distribution may provide a compositional explanation for the apparent age- and sex-adjusted co-detection association and its attenuation after adenovirus-positive episodes were excluded. Based on this apparent age- and sex-adjusted co-detection association, leave-one-virus-out analyses were performed to assess whether the estimate was disproportionately influenced by specific viral targets. Analyses excluding influenza A, influenza B, RSV A, RSV B, human metapneumovirus, parainfluenza virus types 1–3, rhinovirus, coronavirus 229E, OC43, NL63, enterovirus, and bocavirus each showed estimates that were broadly similar in direction. In contrast, when adenovirus-positive episodes were excluded, the association was attenuated to 2.7% (95% CI, −5.8 to 12.0; *p* = 0.541), whereas exclusion of rhinovirus strengthened the association to 19.8% (95% CI, 7.7–33.4; *p* < 0.001) (Table 2).

In an additional full-period clustering sensitivity analysis restricted to the 12 viral targets available throughout the analytical period, 9989 virus-positive episodes from 8528 patients were included. The adjusted co-detection estimate was not statistically significant after accounting for within-patient clustering and viral composition (+7.6%; 95% CI, −12.8 to 32.7; *p* = 0.496). After adenovirus-positive episodes were excluded, 8155 episodes from 7125 patients remained, and the co-detection estimate was also statistically nonsignificant (+12.1%; 95% CI, −22.0 to 61.2; *p* = 0.536) (Table S5).

**Figure 3:**
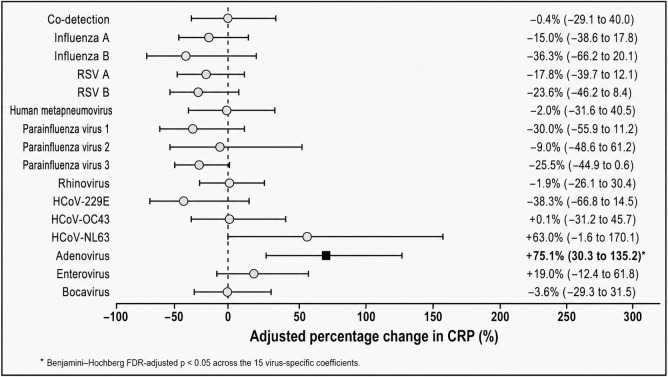
Adjusted associations of co-detection status and virus-specific detection indicators with C-reactive protein levels during the complete-panel period. Forest plot showing adjusted percentage changes in C-reactive protein (CRP) associated with viral co-detection status and individual respiratory viral targets in the complete-panel mixed-effects model. The analysis included 1839 virus-positive episodes from 1636 patients collected from 25 June 2018 through 30 December 2024. The model included age group, sex, co-detection status, and all 15 virus-specific detection indicators as fixed effects, with a patient-level random intercept. Points represent adjusted percentage changes in CRP, and horizontal lines represent 95% confidence intervals. The dashed vertical line at 0% indicates no adjusted difference in CRP. The asterisk indicates statistical significance after Benjamini–Hochberg correction across the 15 virus-specific coefficients.

Because respiratory virus target availability changed during the study period, the complete-panel mixed-effects analysis was restricted to virus-positive episodes collected from 25 June 2018 through 30 December 2024. The analysis included 1839 episodes from 1636 unique patients, comprising 1345 single-virus detection episodes and 494 co-detection episodes. Compared with infants aged <1 year, adjusted CRP levels were 87.1% higher among children aged 1–12 years (95% CI, 57.2–122.6), 196.4% higher among adolescents aged 13–18 years (95% CI, 78.0–393.5), 907.1% higher among adults aged 19–64 years (95% CI, 669.0–1218.8), and 1914.9% higher among adults aged ≥65 years (95% CI, 1506.4–2427.4); all *p*-values were <0.001. After simultaneous adjustment for age group, sex, viral composition, and repeated measurements within patients, co-detection was not independently associated with CRP (−0.4%; 95% CI, −29.1 to 40.0; *p* = 0.982). Adenovirus was associated with a 75.1% higher adjusted CRP level (95% CI, 30.3–135.2; *p* < 0.001) and remained significant after FDR correction across the 15 virus-specific coefficients (FDR-adjusted *p* = 0.003) (Figure 3; Table 3). No other virus-specific indicator was statistically significant in the complete-panel model. Full model estimates are provided in Table S4.

Thus, the lower median CRP observed in adenovirus-containing co-detection episodes than in adenovirus single-virus detections should not be interpreted as inconsistent with the positive adjusted adenovirus estimate. The former represents a within-virus descriptive comparison by detection status, whereas the latter estimates the independent association of adenovirus presence across virus-positive episodes after accounting for co-detection status and covariates. Additional sensitivity analyses accounting for assay-platform differences and the COVID-19 pandemic period yielded results consistent with the primary composition-adjusted analyses. In the full-period GEE model, additionally adjusted for assay platform and pandemic period, co-detection was not independently associated with CRP (+6.5%; 95% CI, −13.6 to 31.4; *p* = 0.553). The association also remained nonsignificant when the analysis was restricted to the AdvanSure platform period (+0.7%; 95% CI, −22.5 to 30.8; *p* = 0.960) and when the pandemic period was added to the complete-panel mixed-effects model (+0.7%; 95% CI, −28.2 to 41.4; *p* = 0.966) (Table S7).

## 4. Discussion

The principal finding of this study was that the age- and sex-adjusted association between viral co-detection and CRP was not retained after simultaneous adjustment for viral composition and repeated measurements within patients. Although co-detection was associated with an 11.8% higher CRP level after adjustment for age and sex alone, the corresponding estimate was −0.4% in the complete-panel mixed-effects model. Age group showed strong graded associations with CRP, whereas adenovirus was the only virus-specific indicator independently associated with higher CRP after full adjustment. These age-group estimates represent composite demographic associations that may encompass age-related biological, clinical, and exposure-related factors. These findings indicate that the detected target number alone did not explain concurrent CRP profiles and support an interpretive framework that prioritizes patient age and the specific viral constituents of a co-detection episode. These absolute CRP concentrations represent continuous same-day laboratory profiles in an age-heterogeneous cohort. These values should be interpreted in relation to patient age, laboratory context, and viral composition, whereas assessment of systemic inflammation requires consideration of the broader clinical and laboratory context. Accordingly, the present study characterizes CRP-profile heterogeneity rather than categorical SIR status.

At the individual-patient level, the same-day CRP concentration may also reflect the intensity and anatomical extent of inflammation, host characteristics, and the stage of infection. Accordingly, the detected target number was interpreted as a group-level laboratory characteristic rather than as a direct measure of inflammatory severity in an individual patient. This framework is consistent with the biological diversity of respiratory viruses in terms of replication kinetics, tissue tropism, and innate immune activation, and with the heterogeneous significance of viral co-detection across populations and viral combinations [12,13,14]. Directional differences between the descriptive, within-virus comparisons, and the mixed-effects estimates, particularly for adenovirus, likely reflect differences in estimands rather than contradictory findings. Specifically, the lower median CRP observed in adenovirus-containing co-detection episodes compared with adenovirus single-virus detections does not negate the positive adjusted association of adenovirus presence with CRP across virus-positive episodes. The descriptive analyses compared CRP between single-virus detection and co-detection within each virus, whereas the mixed-effects model estimated adjusted virus-specific associations with CRP relative to episodes without that virus after accounting for co-detection status and other covariates. The more reproducible finding was composition-dependent heterogeneity, indicating that the same-day CRP profile of a co-detection episode may depend more on the specific viral constituents involved than on the number of detected viruses. This pattern is biologically plausible, but it should be interpreted cautiously because PCR-based co-detection does not necessarily indicate simultaneous active replication of all detected viral targets. Different viral combinations may be associated with distinct host-response profiles, and prior meta-analytical evidence indicates that co-detection is not consistently associated with increased clinical severity. Previous mechanistic and epidemiological studies of viral interference and respiratory viral co-detection provide a biologically plausible basis for the hypothesis that virus–virus interactions may influence non-additive host-response patterns, although these interactions could not be directly tested in the present laboratory-based dataset [15,16,17].

From a virological perspective, the observed composition-dependent CRP patterns are consistent with the concept that respiratory viral co-detection is not necessarily additive. However, these patterns should be interpreted as statistical associations rather than as direct evidence that viral co-presence biologically amplifies or attenuates host inflammatory responses. Several non-mutually exclusive mechanisms may contribute to the observed heterogeneity. Viral interactions, including interference, may modulate host inflammatory signaling during co-detection episodes [18,19,20]. Differential viral load may also contribute, because one or more targets in a co-detection episode may represent low-level detection, residual shedding, or sequential infection, whereas higher viral loads have been associated with more pronounced clinical phenotypes [21,22]. The timing of presentation is relevant because CRP values generally increase during the early phase of illness; therefore, differences in the timing of sample collection relative to symptom onset could affect same-day CRP values, independent of the eventual inflammatory trajectory [23,24,25,26].

Of the individual viruses, adenovirus was the most prominent contributor to composition-dependent CRP heterogeneity. Adenovirus was markedly over-represented among co-detection episodes, and its presence was independently associated with a 75.1% higher CRP level in the complete-panel model. Furthermore, exclusion of adenovirus-positive episodes reduced the age- and sex-adjusted co-detection estimate from 11.8% to 2.7%, with the confidence interval including the null value. These findings suggest that the apparent association between co-detection and higher CRP was substantially influenced by the viral composition of the co-detection group rather than by co-detection status itself. Adenoviral respiratory infection, particularly in pediatric patients, has been associated with relatively pronounced systemic inflammatory responses and marked CRP elevation, even in the absence of confirmed bacterial co-detection [27,28,29]. Nevertheless, the present results should not be interpreted as evidence that adenovirus uniformly causes high CRP or accounts for all variation among co-detection episodes. Rather, adenovirus represents a major composition-specific contributor that should be considered when interpreting concurrent CRP profiles.

The primary implication of this study concerns laboratory interpretation. When multiple respiratory viral targets are co-detected, same-day CRP profiles can be more informatively interpreted by considering patient age and the specific viral composition than by relying on target count alone. CRP remains a nonspecific host-response measure and should be used to contextualize, rather than determine, the significance of categorical multiplex PCR results. This framework may provide additional laboratory context when viral-load data, cycle-threshold values, sequencing, or comprehensive clinical adjudication are unavailable; however, it should be interpreted in light of temporal changes in assay platforms and viral target availability. Because clinical outcomes, bacterial co-infection, and antimicrobial prescribing were not evaluated, these findings do not establish effects on clinical management or antimicrobial stewardship.

This study had some limitations. As cycle threshold (Ct) values, symptom timing, bacterial testing, and clinical outcomes were not systematically available, the present findings should be interpreted as laboratory-based associations relevant to multiplex PCR result interpretation, rather than as evidence of active viral co-infection, clinical severity, bacterial co-infection, or causal virus–virus interactions. As information on symptom onset and Ct values was unavailable, the analysis could not adequately account for differences in inflammatory responses according to the stage of infection or viral load [30,31]. In particular, the absence of Ct values prevented distinction between high-replication co-infection, low-level residual shedding, and sequential infection. No viral genomic sequencing data were available; therefore, strain-level variation, lineage replacement, and genotype-specific inflammatory patterns could not be evaluated. Clinical information, including bacterial detection, antibiotic exposure, radiologic findings, disease severity, hospitalization status, and testing indications, was not systematically linked to the dataset, potentially resulting in residual confounding, misclassification, and spectrum bias. The laboratory dataset captured age, sex, viral targets, and CRP, while detailed age-specific host and exposure variables remained outside the analytical scope. In children, genetic background, immune-mediated conditions, and concurrent infections may contribute to CRP variability; in adults, smoking, adiposity, chronic stress, and chronic diseases may contribute; and in older adults, age-related chronic low-grade inflammation may influence both baseline CRP and susceptibility to respiratory viral infection. These factors represent plausible sources of residual heterogeneity within the predefined age groups. In addition, because the analysis included only multiplex PCR episodes with same-day CRP measurements, nonrandom CRP ordering by clinicians may have enriched the analytical cohort for episodes with greater suspected inflammation or bacterial co-infection. Assay platform changes between 2008–2012 and 2013–2024, changes in viral target availability, and pandemic-related changes in respiratory virus epidemiology and testing practice may have influenced co-detection rates and virus-specific composition. However, the co-detection estimate remained statistically nonsignificant in the full-period analysis, additionally adjusted for assay platform and pandemic period; in the AdvanSure-only analysis; and in the complete-panel model additionally adjusted for pandemic period (Table S7). These findings support the robustness of the primary composition-adjusted results to major temporal changes in testing practice. Nevertheless, pandemic-period adjustment should be interpreted as a contextual sensitivity analysis rather than direct adjustment for SARS-CoV-2 infection, because SARS-CoV-2 was not included among the viral targets evaluated in the present dataset. The wide confidence interval surrounding the complete-panel co-detection estimate also indicates limited precision for separating a residual target-count effect from the contributions of individual viruses. In addition, CRP values were capped at the upper reportable limit of 70 mg/dL, which may have attenuated differences driven by the most pronounced inflammatory episodes; however, the use of medians and log-transformed values likely limited the influence of this upper-range constraint. Finally, this study did not directly evaluate downstream clinical outcomes or management decisions after multiplex PCR reporting. Future prospective studies integrating CRP with interleukins, procalcitonin, pancreatic stone protein, other protein-based host-response panels, and quantitative viral-load measures may provide a more comprehensive characterization of host responses and further refine the interpretation of respiratory multiplex PCR results.

## 5. Conclusions

In this 2008–2024 laboratory-based cohort, patient age and viral composition provided more informative context for same-day CRP profiles in respiratory viral co-detection than did the number of detected targets. The age- and sex-adjusted association between co-detection and higher CRP was not retained after adjustment for viral composition and repeated measurements, whereas adenovirus remained independently associated with higher CRP. These findings support the interpretation of same-day CRP profiles according to patient age and the specific viruses detected rather than target count alone.

## Figures and Tables

**Table 1:** Unadjusted comparison of CRP levels between single-virus detection and viral co-detection.

Analysis Item	Single-Virus Detection	Viral Co-Detection	*p*-Value
N	7816	2437	
Median CRP (mg/dL)	0.74	0.79	0.508
IQR	0.22–2.53	0.26–2.48	
Log-transformed regression	Reference	+1.8% change (95% CI, –5.6 to 9.8)	0.639

Abbreviations: CRP, C-reactive protein; IQR, interquartile range; CI, confidence interval. The *p*-value for median CRP was derived from the Wilcoxon rank-sum test; the *p*-value for regression was derived from unadjusted log-transformed linear regression.

**Table 2:** Leave-one-virus-out sensitivity analysis of the association between viral co-detection and log-transformed C-reactive protein (CRP).

Excluded Virus	Remaining N	Change in CRP (%)	95% CI	*p*-Value
None	10,253	11.8	4.3 to 19.9	0.002
Influenza A	9291	13.4	5.4 to 22.1	<0.001
Influenza B	9956	11.4	3.8 to 19.6	0.003
RSV A	8793	10.5	2.5 to 19.2	0.009
RSV B	8995	10.5	2.6 to 19.2	0.009
Human metapneumovirus	9451	12.6	4.6 to 21.1	0.002
Parainfluenza virus 1	9870	10.6	2.9 to 18.7	0.006
Parainfluenza virus 2	10,123	12.6	5.0 to 20.8	<0.001
Parainfluenza virus 3	9349	8.1	0.3 to 16.5	0.041
Rhinovirus	6694	19.8	7.7 to 33.4	<0.001
HCoV-229E	9984	11.7	4.1 to 20.0	0.002
HCoV-OC43	9816	11.6	3.8 to 20.0	0.003
HCoV-NL63	10,127	10.9	3.3 to 19.0	0.004
Adenovirus	8419	2.7	−5.8 to 12.0	0.541
Enterovirus	9974	14.9	6.9 to 23.5	<0.001
Bocavirus	9851	12.3	4.4 to 20.8	0.002

Abbreviations: CRP, C-reactive protein; CI, confidence interval. Percentage changes were derived from linear regression models using log(CRP + 0.01) as the outcome. In each leave-one-virus-out analysis, episodes positive for the indicated virus were excluded using the same virus-specific indicator definitions as those used for the descriptive virus counts in Tables S1 and S2, and the association between co-detection and CRP was re-estimated after adjustment for age group and sex.

**Table 3:** Adjusted mixed-effects model estimates for co-detection status and statistically significant virus-specific indicators during the complete-panel period.

Variable	Adjusted Percentage Change in CRP (%)	95% CI	*p*-Value
Co-detection	−0.4	−29.1 to 40.0	0.982
Adenovirus	75.1	30.3 to 135.2	<0.001

Abbreviations: CRP, C-reactive protein; CI, confidence interval. Percentage changes were estimated using a linear mixed-effects model with log(CRP + 0.01) as the outcome. The model included age group, sex, co-detection status, and all 15 virus-specific detection indicators as fixed effects, with a patient-level random intercept. The analysis included 1839 virus-positive episodes from 1636 patients collected from 25 June 2018 through 30 December 2024, comprising 1345 single-virus detection episodes and 494 co-detection episodes. Co-detection was retained as the primary predictor regardless of statistical significance; among virus-specific indicators, only adenovirus had a nominal *p*-value < 0.05 and remained significant after Benjamini–Hochberg correction across the 15 virus-specific coefficients (FDR-adjusted *p* = 0.003). Full model estimates are provided in Table S4.

## Data Availability

The data analyzed in this study were derived from clinical records at Dankook University Hospital and are subject to ethical and legal restrictions. Owing to patient privacy and confidentiality considerations, the original datasets cannot be made publicly available. De-identified aggregated data may be provided by the corresponding author upon reasonable request, subject to approval by the Institutional Review Board.

## Supplementary Materials

The following supporting information can be downloaded at: https://www.mdpi.com/article/10.3390/microorganisms14071543/s1. Table S1: Baseline characteristics of the study population and distribution of detection categories and respiratory viruses; Table S2: Comparison of median C-reactive protein concentrations between single-virus detections and co-detections for each respiratory virus; Table S3: Most frequent exact viral co-detection combinations and corresponding C-reactive protein distributions; Table S4: Full mixed-effects model estimates for the complete-panel period; Table S5: Full-period generalized estimating equation sensitivity analysis restricted to 12 viral targets available throughout 2008–2024; Table S6. Demographic and calendar-period characteristics according to respiratory multiplex PCR detection category; Table S7. Sensitivity analyses accounting for assay-platform differences and the COVID-19 pandemic period; Figure S1. Median C-reactive protein (CRP) levels by respiratory virus according to detection status: Single-virus detection versus co-detection.

## Abbreviations

The following abbreviations are used in this manuscript:

CRP	C-reactive protein
PCR	Polymerase chain reaction
RSV	Respiratory syncytial virus
RSV A	Respiratory syncytial virus A
RSV B	Respiratory syncytial virus B
IQR	Interquartile range
CI	Confidence interval
FDR	False discovery rate
Ct	Cycle threshold
RV	Respiratory virus
