# Supplementary material for: Age- and Virus-Composition-Aware Interpretation of Respiratory Viral Co-Detection Using Same-Day C-Reactive Protein Profiles: A 2008–2024 Multiplex PCR Laboratory Cohort Study
Source: Microorganisms. 2026 Jul 15;14(7):1543. doi: 10.3390/microorganisms14071543 (PMC13414455; doi:10.3390/microorganisms14071543)
Supplement: Supplementary file 1 [file microorganisms-14-01543-s001.zip › microorganisms-4447338-supplementary.pdf]

Supplementary Material

Age- and Virus-Composition–Aware Interpretation of Respiratory Viral Co-Detection Using Same-Day C-Reactive Protein Profiles: A 2008–2024 Multiplex PCR Laboratory Cohort Study

Sung Hun Jang[1,†], Jeong Su Han[2,†], Bo Kyeung Jung[3], Ga-Yeon Kim[4], Jae Kyung Kim[2,*]

[1] Department of Medical Laser, Graduate School of Medicine, Dankook University, Cheonan-si 31116, Republic of Korea; well8143@naver.com

[2] Department of Biomedical Laboratory Science, College of Health Sciences, Dankook University, Cheonan-si 31116, Republic of Korea; jshan1162@naver.com (J.S. Han)

[3] Department of Laboratory Medicine, College of Medicine, Dankook University, Cheonan-si 31116, Republic of Korea; lovegodmother@hanmail.net (B.K. Jung)

[4] Department of Public Health, Graduate School, Dankook University, Cheonan-si 31116, Chungnam, Republic of Korea; sysnhj77@gmail.com (G.-Y. Kim)

[*]**Correspondence**: nerowolf@naver.com; Tel.: +82-41-550-1451

ORCID: S.H. Jang, 0009-0009-0934-8445; J.S. Han, 0009-0008-7003-8422; B.K. Jung, 0000-0002-9785-6440; G.-Y. Kim, 0000-0001-8751-5055; J.K. Kim, 0000-0002-1534-563X

[†]These authors contributed equally to this work

Table S1. Baseline characteristics of the study population and distribution of detection categories and respiratory viruses.

Variable	Category	n (%) / Value	Denominator
**Study population**	Total episodes	19,002	—
	Age, median (IQR), years	3 (0–44)	—
	Age range, years	0–98	—
	Male	11,338 (59.7)	19,002
	Female	7,664 (40.3)	19,002
**Detection category**	PCR-not-detected	8,749 (46.0)	19,002
	Single-virus detection	7,816 (41.1)	19,002
	Co-detection	2,437 (12.8)	19,002
	Total positive	10,253 (54.0)	19,002
**Virus distribution**	Rhinovirus	3,559 (18.7)	19,002
	Adenovirus	1,834 (9.7)	19,002
	RSV A	1,460 (7.7)	19,002
	RSV B	1,258 (6.6)	19,002
	Influenza A	962 (5.1)	19,002
	Parainfluenza virus 3	904 (4.8)	19,002
	Human metapneumovirus	802 (4.2)	19,002
	OC43	437 (2.3)	19,002
	Bocavirus	402 (4.0)	10,140
	Parainfluenza virus 1	383 (2.0)	19,002
	Influenza B	297 (1.6)	19,002
	Enterovirus	279 (5.0)	5,583
	Coronavirus 229E	269 (1.4)	19,002
	Parainfluenza virus 2	130 (0.7)	19,002
	NL63	126 (1.2)	10,140

Abbreviations: IQR, interquartile range. Virus distribution percentages were calculated using the number of episodes in which each virus was tested during the study period as the denominator. NL63 and bocavirus were evaluated in episodes collected from 12 January 2015 through 30 December 2024 (n = 10,140), and enterovirus was evaluated in episodes collected from 25 June 2018 through 30 December 2024 (n = 5,583). The remaining 12 viral targets were evaluated in all episodes collected from 2 October 2008 through 30 December 2024 (n = 19,002). Virus-specific counts are not mutually exclusive because co-detection episodes contributed to each detected viral target.

Table S2. Comparison of median C-reactive protein concentrations between single-virus detections and co-detections for each respiratory virus.

Virus	Single-virus detection, n	CRP median (IQR), single	Co-detection, n	CRP median (IQR), co-detection	FDR-adjusted *p*-value
Adenovirus	800	1.86 (0.64–4.75)	1,034	1.09 (0.35–3.05)	<0.001
Influenza A	762	1.50 (0.41–5.43)	200	0.92 (0.21–3.07)	0.005
RSV B	887	0.44 (0.10–1.52)	371	0.58 (0.19–2.07)	0.008
Parainfluenza virus 1	245	0.43 (0.10–1.34)	138	0.78 (0.27–2.18)	0.015
Enterovirus	69	0.90 (0.36–3.63)	210	0.56 (0.22–1.92)	0.015
Parainfluenza virus 3	528	0.52 (0.15–1.88)	376	0.80 (0.25–2.46)	0.015
RSV A	1,097	0.35 (0.07–1.20)	363	0.44 (0.14–1.42)	0.015
Human metapneumovirus	541	1.01 (0.34–3.02)	261	0.82 (0.28–2.48)	0.236
Rhinovirus	2,031	0.77 (0.28–2.33)	1,528	0.73 (0.25–2.34)	0.385
Bocavirus	124	0.55 (0.13–2.13)	278	0.68 (0.22–2.42)	0.464
HCoV-229E	157	0.99 (0.34–3.42)	112	0.94 (0.30–2.86)	0.464
HCoV-NL63	62	0.69 (0.30–2.18)	64	1.00 (0.38–3.16)	0.540
HCoV-OC43	208	0.60 (0.17–4.03)	229	0.78 (0.20–2.25)	0.713
Influenza B	233	0.60 (0.22–2.48)	64	0.85 (0.22–1.91)	0.985
Parainfluenza virus 2	72	0.50 (0.23–1.38)	58	0.52 (0.16–1.66)	0.998

Abbreviations: CRP, C-reactive protein; IQR, interquartile range

P-values were adjusted for multiple comparisons across the 15 viruses using the Benjamini–Hochberg false discovery rate (FDR) procedure.

Table S3. Most frequent exact viral co-detection combinations and corresponding C-reactive protein distributions.

Rank	Viral combination	Detected targets	Episodes, n	Median CRP, mg/dL	IQR, mg/dL
1	Rhinovirus + Adenovirus	2	374	1.36	0.42–3.35
2	Parainfluenza virus 3 + Rhinovirus	2	136	0.63	0.11–1.72
3	Rhinovirus + Enterovirus	2	129	0.52	0.20–1.99
4	RSV A + Rhinovirus	2	127	0.37	0.15–1.05
5	RSV B + Rhinovirus	2	127	0.41	0.11–1.56
6	RSV B + Adenovirus	2	91	0.84	0.33–2.98
7	Human metapneumovirus + Rhinovirus	2	80	0.87	0.31–2.16
8	Rhinovirus + Bocavirus	2	71	0.53	0.18–1.37
9	Parainfluenza virus 1 + Rhinovirus	2	65	0.82	0.27–2.47
10	RSV A + Adenovirus	2	60	0.80	0.28–2.73
11	Human metapneumovirus + Adenovirus	2	49	1.16	0.44–2.71
12	Parainfluenza virus 3 + Adenovirus	2	47	1.36	0.54–3.18
13	Influenza A + Adenovirus	2	37	1.18	0.31–2.93
14	Influenza A + Rhinovirus	2	37	0.66	0.10–1.97
15	HCoV-OC43 + Adenovirus	2	35	1.06	0.27–2.57
16	Adenovirus + Bocavirus	2	32	1.66	0.75–3.46
17	RSV A + HCoV-OC43	2	32	0.35	0.07–1.67
18	Parainfluenza virus 3 + Rhinovirus + Adenovirus	3	31	1.52	0.28–3.50
19	Rhinovirus + HCoV-OC43	2	29	0.59	0.24–2.83
20	Parainfluenza virus 3 + Bocavirus	2	28	0.49	0.19–1.53

Co-detection combinations were defined according to the exact set of virus-specific positive indicators detected in each episode. All 2,437 co-detection episodes were assigned to exact viral-combination categories before ranking. Table S3 displays the 20 most frequent combinations, which together accounted for 1,617 of 2,437 co-detection episodes (66.4%). CRP values are presented as medians and interquartile ranges.

Table S4. Full mixed-effects model estimates for the complete-panel period.

Variable	Adjusted % change in CRP	95% CI	p-value
Co-detection	−0.4	−29.1 to 40.0	0.982
Age 1–12 years	87.1	57.2 to 122.6	<0.001
Age 13–18 years	196.4	78.0 to 393.5	<0.001
Age 19–64 years	907.1	669.0 to 1218.8	<0.001
Age ≥ 65 years	1914.9	1506.4 to 2427.4	<0.001
Male sex	−3.6	−16.1 to 10.9	0.610
Influenza A	−15.0	−38.6 to 17.8	0.330
Influenza B	−36.3	−66.2 to 20.1	0.164
RSV A	−17.8	−39.7 to 12.1	0.216
RSV B	−23.6	−46.2 to 8.4	0.131
Human metapneumovirus	−2.0	−31.6 to 40.5	0.913
Parainfluenza virus 1	−30.0	−55.9 to 11.2	0.131
Parainfluenza virus 2	−9.0	−48.6 to 61.2	0.747
Parainfluenza virus 3	−25.5	−44.9 to 0.6	0.055
Rhinovirus	−1.9	−26.1 to 30.4	0.896
HCoV-229E	−38.3	−66.8 to 14.5	0.126
HCoV-OC43	0.1	−31.2 to 45.7	0.996
HCoV-NL63	63.0	−1.6 to 170.1	0.058
Adenovirus	75.1	30.3 to 135.2	<0.001
Enterovirus	19.0	−12.4 to 61.8	0.266
Bocavirus	−3.6	−29.3 to 31.5	0.816

Percentage changes were estimated using a linear mixed-effects model with log(CRP + 0.01) as the outcome. The model included co-detection status, age group, sex, and all 15 virus-specific indicators as fixed effects, with a patient-level random intercept. The analysis was restricted to 1,839 virus-positive episodes from 1,636 unique patients collected from 25 June 2018 through 30 December 2024, including 1,345 single-virus detection episodes and 494 co-detection episodes. Virus-specific estimates represent adjusted associations compared with episodes without the corresponding virus. P-values were calculated using the Wald normal approximation. Among the 15 virus-specific coefficients, only adenovirus remained statistically significant after Benjamini–Hochberg FDR correction (FDR-adjusted $p = 0.003$).

Table S5. Full-period generalized estimating equation sensitivity analysis restricted to 12 viral targets available throughout 2008–2024.

Model	Episodes, n	Unique patients, n	Single-virus detection, n	Co-detection, n	Adjusted CRP change (%)	95% CI	p-value
Full-period GEE, 12 constant targets	9,989	8,528	7,922	2,067	7.6	−12.8 to 32.7	0.496
Full-period GEE excluding adenovirus-positive episodes	8,155	7,125	7,069	1,086	12.1	−22.0 to 61.2	0.536

Abbreviations: CI, confidence interval; CRP, C-reactive protein; GEE, generalized estimating equation. Percentage changes were estimated using GEE models with log(CRP + 0.01) as the outcome, an exchangeable working correlation structure, and patient identifier as the clustering variable. The analysis was restricted to virus-positive episodes defined using the 12 viral targets available throughout 2008–2024. Models included co-detection status, age group, sex, and virus-specific indicators.

Table S6. Demographic and calendar-period characteristics according to respiratory multiplex PCR detection category.

Variable	PCR-not-detected	Single-virus detection	Viral co-detection
Episodes, n	8,749	7,816	2,437
Age, median (IQR), years	18 (1–70)	1 (0–5)	1 (0–3)
<1 year, n (%)	1,833 (21.0)	2,925 (37.4)	748 (30.7)
1–12 years, n (%)	2,198 (25.1)	3,646 (46.6)	1,563 (64.1)
13–18 years, n (%)	347 (4.0)	184 (2.4)	16 (0.7)
19–64 years, n (%)	1,753 (20.0)	482 (6.2)	53 (2.2)
≥65 years, n (%)	2,618 (29.9)	579 (7.4)	57 (2.3)
Male sex, n (%)	5,391 (61.6)	4,475 (57.3)	1,472 (60.4)
Female sex, n (%)	3,358 (38.4)	3,341 (42.7)	965 (39.6)
2008–2012, n (%)	2,161 (24.7)	2,994 (38.3)	784 (32.2)
2013–2017, n (%)	2,609 (29.8)	3,173 (40.6)	1,074 (44.1)
2018–2024, n (%)	3,979 (45.5)	1,649 (21.1)	579 (23.8)

The 2018–2024 calendar-period category includes all episodes collected from 1 January 2018. In contrast, the complete-panel mixed-effects analysis began on 25 June 2018, when results for all 15 respiratory viral targets became available in the analytical dataset. **Abbreviations:** IQR, interquartile range; PCR, polymerase chain reaction

Table S7. Sensitivity analyses accounting for assay-platform differences and the COVID-19 pandemic period.

Analysis	Episodes, n	Unique patients, n	Single-virus detection, n	Co-detection, n	Adjusted CRP change, %	95% CI	p-value
Full-period GEE, 12 consistently available targets; adjusted for assay platform and pandemic period	9,989	8,528	7,922	2,067	6.5	−13.6 to 31.4	0.553
AdvanSure platform period only; adjusted for pandemic period	6,211	5,295	4,928	1,283	0.7	−22.5 to 30.8	0.960
Complete-panel mixed-effects model; additionally adjusted for pandemic period	1,839	1,636	1,345	494	0.7	−28.2 to 41.4	0.966

Abbreviations: CI, confidence interval; CRP, C-reactive protein; GEE, generalized estimating equation. Percentage changes were derived from models using log(CRP + 0.01) as the outcome. The full-period GEE model included co-detection status, age group, sex, assay platform, pandemic period, and indicators for the 12 viral targets available throughout 2008–2024, with patient identifier as the clustering variable. The AdvanSure-only model was restricted to episodes collected from 2013 through 2024 and included co-detection status, age group, sex, pandemic period, and indicators for the same 12 viral targets. The complete-panel mixed-effects model included age group, sex, co-detection status, pandemic period, all 15 virus-specific indicators, and a patient-level random intercept. Pandemic periods were defined as pre-pandemic (2008–2019), pandemic (2020–2022), and post-pandemic (2023–2024).

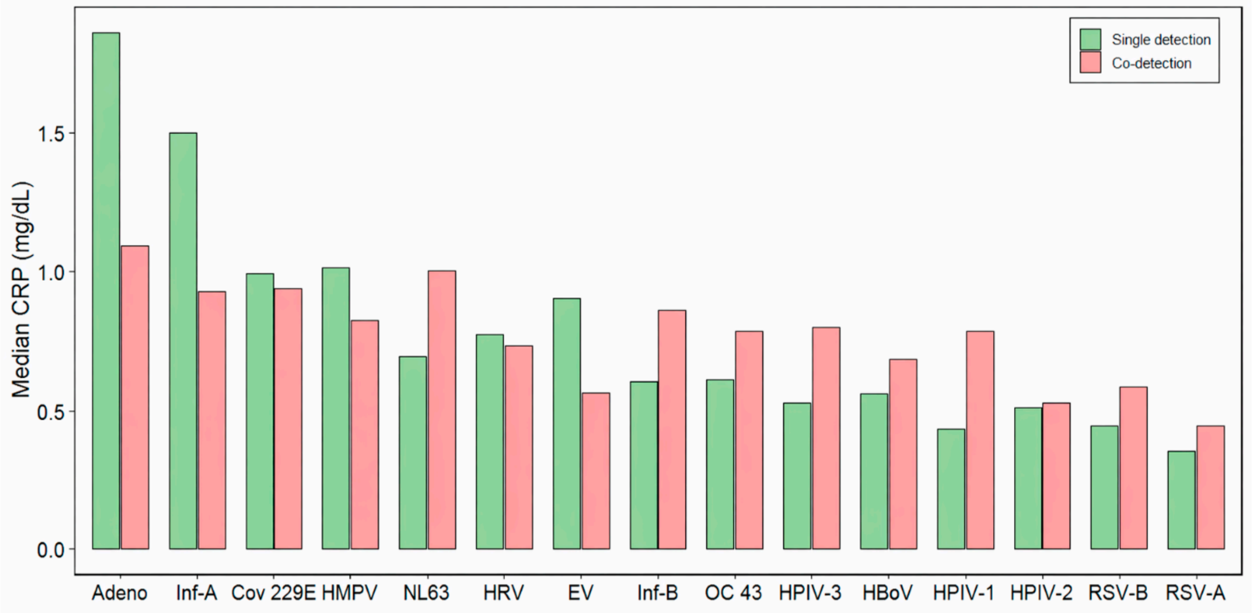

Figure S1. Median C-reactive protein (CRP) levels by respiratory virus according to detection status: Single-virus detection versus co-detection. Bars represent the median CRP level (mg/dL) for each virus in episodes of single-virus detection and co-detection. For each virus, a higher bar indicates a higher median CRP concentration, used here as a nonspecific concurrent host-response measure. The direction and magnitude of CRP differences varied across viruses, supporting composition-dependent rather than uniform interpretation of viral co-detection. Abbreviations: Adeno, adenovirus; Inf-A, influenza A; HMPV, human metapneumovirus; HRV, rhinovirus; EV, enterovirus; HBoV, human bocavirus; HPIV, human parainfluenza virus; RSV, respiratory syncytial virus
